# Body mass index and healthy lifestyle practices among Peruvian university students: a comparative study among academic discipline

**DOI:** 10.3389/fnut.2024.1361394

**Published:** 2024-02-21

**Authors:** Jacksaint Saintila, Yaquelin E. Calizaya-Milla, Sandra P. Carranza-Cubas, Antonio Serpa-Barrientos, Susan M. Oblitas-Guerrero, Cristian Ramos-Vera

**Affiliations:** ^1^Escuela de Medicina Humana, Universidad Señor de Sipán, Chiclayo, Peru; ^2^Research Group for Nutrition and Lifestyle, Universidad Peruana Unión, Lima, Peru; ^3^Departamento de Psicología, Universidad Nacional Mayor de San Marcos, Lima, Peru; ^4^Escuela de Enfermería, Universidad Señor de Sipán, Chiclayo, Peru; ^5^Área de Investigación, Universidad César Vallejo, Lima, Peru

**Keywords:** body mass index, noncommunicable diseases, obesity, healthy lifestyle, universities

## Abstract

**Background:**

Excess body weight and an unhealthy lifestyle are a risk factor for noncommunicable diseases. University students are susceptible to unhealthy habits and obesity. This study compared body mass index (BMI) and healthy lifestyle practices among university students from four academic disciplines: Health Sciences, Business Sciences, Human Sciences and Education, and Engineering/Architecture.

**Methods:**

A cross-sectional study was carried out using a sample of 6,642 university students selected by non-probability convenience sampling. The Diet and Healthy Lifestyle Scale (DEVS), the Peruvian validation of the Vegetarian Lifestyle Index (VLI), was used to assess healthy lifestyle practices.

**Results:**

Students in the areas of Business Sciences and Engineering/Architecture had a higher BMI than their peers in Health Sciences (B = 0.35, 95% CI: 0.15–0.56 and 0.32, 95% CI: 0.13–0.52; *p* = 0.001). Additionally, these students tended to adopt less healthy lifestyle (B = −0.11, 95% CI: −0.20 to −0.01 and −0.09, 95% CI: −0.18 to −0.00; *p* < 0.05) compared to those in Health Sciences.

**Conclusion:**

Although students of Health Sciences and Human Sciences and Education exhibited healthy lifestyle patterns, there is a clear need to improve eating and living habits in general among the university population to mitigate the risk factors associated with non-communicable diseases.

## Introduction

Body mass index (BMI) is defined as a measure that compares an individual’s weight in relation to height; it is calculated by dividing weight in kilograms by the square of height in meters (kg/m^2^) (1). BMI is widely used to classify individuals into different weight categories, which helps identify potential health risks associated with underweight and excess body weight (overweight/obesity) (2). Excess body weight in young people, particularly among university students, represents one of the most important public health problems worldwide. In Peru, according to actual data from the National Institute of Statistics and Informatics (INEI), 37.5% and 25.6% of individuals aged 15 years and older were overweight and obese, respectively (3); the urban area and women are the most affected by obesity, with a prevalence of 39.0% and 29.8%, respectively (3). These statistics position Peru as one of the countries with the highest prevalence of overweight and obesity in the South American region.

Obesity in the university population is a complex phenomenon influenced by multiple factors (4, 5). The most common causes of obesity include unhealthy eating habits, lack of physical activity, academic stress, irregular schedules, lack of time, consumption of harmful substances (tobacco and alcohol), and genetic factors (6–9). Some studies suggest differences in the prevalence of obesity by field of study. For example, a cross-sectional survey conducted on 584 participants reported that Science and Technology students had the highest proportion of overweight and obese individuals compared to those in Health Sciences (10). It is possible that students in disciplines related to health, such as human nutrition, medicine, and nursing, have better lifestyle habits compared to their peers in other disciplines due to their basic knowledge of healthy lifestyles, which could be attributed to greater health awareness and knowledge (11).

A healthy lifestyle is defined as a set of daily behaviors and choices that contribute to an individual’s well-being and optimal health (12). These behaviors include a variety of practices and habits such as healthy eating and nutrition, regular physical activity, adequate water consumption, exposure to sunlight, adequate sleep, among others (13). These habits are related to different aspects of the physical, mental, and social health of university students (14). However, adhering to a healthy lifestyle in a university setting can be difficult for students due to the conditions and characteristics of the environment (15).

The intake of healthy foods, as a component of a healthy lifestyle, is considerably low among young people. In the Peruvian context, according to INEI, only 10.5% of the population over 15 years of age reaches the recommended consumption of at least 5 servings of fruits and vegetables per day (16). In addition, the average daily consumption of fruit in this age group is only 2.0 servings (16), which is below the guidelines established by the World Health Organization (WHO), which recommends consuming 5 or more servings of fruits and vegetables daily to maintain a balanced diet and prevent health conditions such as obesity (17). In addition, it has been observed that no region of the country reaches the ideal average fruit consumption (16). In contrast, both in the general population and in university students, a high consumption of added sugars, processed meats, and saturated fats has been detected, exceeding the daily amounts recommended by the WHO (18). Moreover, the intake of healthy foods has been the subject of studies in several countries (6, 10, 14). In a recent study among university students in Saudi Arabia, only 16.07% and 11.23% of 454 students consumed vegetables and fruits daily, respectively (14). Likewise, another study reported that 47.1% of Science and Technology students consumed meat almost every day (10). This trend in eating habits reflects a significant gap between current dietary practices and national and international nutritional recommendations, underscoring the need to implement strategies to promote healthy eating habits, especially among university students.

Moreover, university students, compared to the general adult population, have reported lower levels of regular physical activity (10, 19–21). The prevalence of physical activity among Peruvian adults is a topic of interest in the field of public health. According to the National Health Survey (ENAHO), 61.9% of this population does not meet international physical activity recommendations (22). Specifically, WHO suggests a minimum of 150 min of moderate physical activity per week for adults (23). Several studies have reported that more than 70% of university students do not reach the recommended goal of 10,000 steps per day (24), This could be due to lack of time, academic factors, among others.

On the other hand, although water is an important chemical element in the body, low water intake is one of the most common health concerns affecting university students (25–27). A study conducted with university students in the United States revealed that only 16.3% of women and 13.3% of men met the recommended daily intake of water (25). Concerns arise when considering these low levels of water intake among university students, especially given the critical relevance of water to various physiological functions of the body. Water is essential for the regulation of body temperature, the effective transport of nutrients through the body, and the proper elimination of waste and toxins, all of which are essential for maintaining good general health (28). Furthermore, adequate sunlight exposure as a healthy lifestyle factor is essential for an individual’s health and well-being (29). University students, particularly those in the field of Health Sciences field, have reported lower levels of sunlight exposure (30). Study in Sri Lanka revealed limited sunlight exposure among university health care students (30). In addition, the influence of modern urban lifestyles, prevalent in this population, has been identified as one of the causes of reduced sunlight exposure (31). Factors such as poor knowledge about the importance of vitamin D, spending long hours in academic facilities such as laboratories, and predominantly sedentary study habits contribute to this low sunlight exposure in students (30, 32). However, it is important to maintain a balance between the benefits of sunlight exposure and the risk of skin damage, including skin cancer (33). This highlights the importance of implementing public health strategies focused on promoting an appropriate balance, optimizing the benefits of sunlight while minimizing the risks associated with excessive exposure.

Despite the growing evidence on the importance of a healthy lifestyle and its impact on student well-being, there is a notable lack of research comparing these variables between different academic disciplines. Several studies have examined the BMI and lifestyles of university students (4, 11, 34, 35), however, most focus on the difference in gender and years of study and do not make clear distinctions between academic disciplines. This lack of specificity prevents a complete understanding of how different fields of study can influence students’ health and behavior. Understanding these specific disparities is important, as it provides a unique perspective on health determinants in university settings and offers the opportunity to develop more effective targeted interventions and public health policies. The identification of specific healthy lifestyle patterns and associated health risks in different academic areas not only underscores the need for personalized health promotion strategies but could also contribute to the prevention of noncommunicable diseases among the university population, a key demographic group in the formation of future generations and in the promotion of healthy lifestyles in society. Therefore, this study aims to investigate the differences in BMI and healthy lifestyle practices among students from different disciplines at a private university in Peru.

## Materials and methods

### Design and participants of the study

This cross-sectional comparative study was carried out at a private Peruvian university with campuses in the three main geographical regions of the country: coast, highlands, and jungle. This purposeful selection allows for a broad cultural and socioeconomic diversity inherent to these regions, reflecting a wider spectrum of the Peruvian student population. The inclusion of campuses in these geographically and culturally distinct areas offers a unique opportunity to examine BMI and healthy lifestyle practices among university students in different environmental and cultural contexts. Data collection was carried out during the enrollment period for the first academic cycle of the year, in the months of February and March 2021. A non-probability convenience sampling method was used to recruit participants. The invitation to students to participate in the study was made through the university’s academic portal, where detailed information on the objectives of the study was provided. This information was available on the survey’s homepage. Subsequently, electronic informed consent was obtained from the students who chose to participate. In total, 6,642 students completed the survey. Adult students between 18 and 29 years of age were considered eligible for the study. We excluded those who did not meet the age criteria, graduate students, those who provided inadequate responses to specific survey questions, and those who did not give their consent. The project was reviewed and approved by the Research Ethics Committee of the Universidad Peruana Unión (approval number: 2021-CEUPeU-0009), and the corresponding permission was obtained from the academic area of the university. The study was carried out according to the ethical standards established in the Declaration of Helsinki and its amendments.

### Variables of study

#### Body mass index

As part of the study, data on the weight and height of the students were collected, which were self-reported by the participants. Using this information, the BMI of each student was calculated. The classification of body mass index (BMI) was performed according to WHO criteria, which define the following categories: (a) underweight, with a BMI of less than 18.5; (b) normal weight, with a BMI between 18.5 and 24.9 kg/m^2^; (c) overweight, with a BMI between 25.0 and 29.9 kg/m^2^; and (d) obese, with a BMI of 30 or more (36).

#### Healthy lifestyle practices

To assess healthy lifestyle practices, we used the Diet and Healthy Lifestyle Scale (DEVS) (37). This instrument represents the validated Peruvian version of the Vegetarian Lifestyle Index (VLI) (38), which consists of 14 items, 11 of which include topics related to whole foods of plant origin, such as fruits, vegetables, legumes, nuts, seeds, and whole grains. Similarly, foods of animal origin, such as milk and dairy products, eggs, and foods that are reliable sources of vitamin B-12, were considered. Sweets were also considered. Additionally, the last 3 items represent lifestyle characteristics and include regular physical activity, adequate water intake, and moderate sunlight exposure. For each question, the response options were limited to 3. The 14 items are summed to obtain a total score ranging from 0 to 14 points, considering the following scoring system: 0, 0.5, or 1 point. Participants were assigned a score based on how well they followed the recommendations: They were awarded 1 point for full compliance, 0.5 points for partial compliance, and 0 points if there was no compliance. For example, participants who consumed ≥6 servings/day of whole grains received a score of 1, those who consumed ≥3 and < 6 servings/day received a score of 0.5; and if they consumed less than 3 servings/day of whole grains they received a score of 0. In addition, inverse scoring was applied for the following components: vegetable oils, dairy products, eggs, sweets, and consumption of foods of animal origin, whose recommendations were to consume in moderation or in moderate amounts, so that higher intakes of these foods received lower scores. For example, participants who consumed >5 servings/week of sweets received 0 points. Those who consumed >2 and ≤ 5 servings/week received 0.5 points. While those who consumed 0 to 2 servings per week received 1 point. On the other hand, in terms of lifestyle variables, we considered the following: For sunlight exposure: <5 min/day = 0 point (low), ≥5 and <10 min/day = 0.5 points (medium), and ≥10 min/day = 1 point (high). Water intake: <4 glasses of water/day = 0 point (low), ≥4 and <8 glasses of water/day = 0.5 points (medium), and ≥8 glasses of water/day = 1 point (high). Daily exercise: 0 min/day of any moderate or vigorous exercise = 0 point (low), >0 and <30 min/day of moderate exercise or >0 and <15 min/day of vigorous exercise, and ≥30 min/day of moderate exercise or ≥15 min/day of vigorous exercise = 1 point (high). Higher total scores indicate a healthy lifestyle (Appendix A) (38, 39).

#### Sociodemographic information

Information was collected based on 8 questions considering the following categories: sociodemographic data, including information on age (years), sex, region of origin, place of residence, marital status, and religion. In addition, academic discipline and the level of parental education were considered.

### Statistical analysis

The descriptive analysis consisted of the description of the variables with absolute and relative frequencies for the categorical variables and mean with standard deviation for the numerical variables. Subsequently, we compare the general characteristics, BMI, and healthy lifestyle according to academic disciplines. We used the Kruskal-Wallis test and the independence chi-square test of to assess whether there are statistically significant differences in the independent numerical and categorical variables, respectively. Finally, we created simple and multiple robust variance linear regression models to evaluate the association between lifestyle and anthropometric parameters with academic disciplines. We considered a value of p of less than 0.05 and the analysis was performed with the R program version 4.3.2.

## Results

Table 1 presents the sociodemographic data of university students divided by academic discipline. The total sample is 6,642 students. The average age is 21.4 years. There is a slight majority of females (54.4%) in the total student population. The students come mainly from the highlands (54.6%). The Faculty of Human Sciences and Education has the highest proportion of students from the Coast (35.8%). Most of the students live in urban areas (71.5%), with a similar proportion in all academic disciplines. Most are Adventists (54.8%), followed by Catholics (33.4%). Most of the students are single (94.4%). Most of the students’ parents have basic education (57.2%).

**Table 1:** General characteristics of Peruvian university students according to academic disciplines.

Characteristics	All	Academic disciplines				
	*N* = 6, 642^a^	Health Sciences	Business Sciences	Human Sciences and Education	Engineering/Architecture	*p* ^b^
		*N* = 2,135^a^ (32.1%)	*N* = 1, 748^a^ (26.3%)	*N* = 715^a^ (10.8%)	*N* = 2,044^a^ (30.8%)	
Age	21.4 (3.4)	21.2 (3.3)	21.5 (3.7)	22.5 (4.1)	21.1 (2.9)	<0.001
Age group (years)						<0.001
≥18	1,075 (16.2%)	376 (17.6%)	274 (15.7%)	89 (12.4%)	336 (16.4%)	
19–24	4,580 (69.0%)	1,479 (69.3%)	1,197 (68.5%)	444 (62.1%)	1,460 (71.4%)	
>24	987 (14.9%)	280 (13.1%)	277 (15.8%)	182 (25.5%)	248 (12.1%)	
Sex						<0.001
Female	3,613 (54.4%)	1,594 (74.7%)	1,003 (57.4%)	369 (51.6%)	647 (31.7%)	
Male	3,029 (45.6%)	541 (25.3%)	745 (42.6%)	346 (48.4%)	1,397 (68.3%)	
Region of origin						<0.001
Coast	1,455 (21.9%)	502 (23.5%)	280 (16.0%)	256 (35.8%)	417 (20.4%)	
Jungle	1,404 (21.1%)	359 (16.8%)	478 (27.3%)	95 (13.3%)	472 (23.1%)	
Highlands	3,627 (54.6%)	1,205 (56.4%)	978 (55.9%)	333 (46.6%)	1,111 (54.4%)	
Foreign	156 (2.3%)	69 (3.2%)	12 (0.7%)	31 (4.3%)	44 (2.2%)	
Lugar de residencia						0.166
Rural	1,886 (28.4%)	589 (27.6%)	518 (29.6%)	220 (30.8%)	559 (27.3%)	
Urbano	4,756 (71.6%)	1,546 (72.4%)	1,230 (70.4%)	495 (69.2%)	1,485 (72.7%)	
Religion						<0.001
Seventh-day Adventist	3,643 (54.8%)	1,254 (58.7%)	786 (45.0%)	522 (73.0%)	1,081 (52.9%)	
Baptist	370 (5.6%)	107 (5.0%)	117 (6.7%)	29 (4.1%)	117 (5.7%)	
Catholic	2,220 (33.4%)	670 (31.4%)	718 (41.1%)	126 (17.6%)	706 (34.5%)	
Others	779 (11.7%)	104 (4.9%)	127 (7.3%)	38 (5.3%)	140 (6.8%)	
Marital status						<0.001
Married	375 (5.6%)	105 (4.9%)	131 (7.5%)	66 (9.2%)	73 (3.6%)	
Single	6,267 (94.4%)	2,030 (95.1%)	1,617 (92.5%)	649 (90.8%)	1,971 (96.4%)	
Parental education						<0.001
Basic	3,801 (57.2%)	1,173 (54.9%)	1,094 (62.6%)	402 (56.2%)	1,132 (55.4%)	
Technical	1,182 (17.8%)	366 (17.1%)	302 (17.3%)	126 (17.6%)	388 (19.0%)	
Undergraduate	1,023 (15.4%)	371 (17.4%)	218 (12.5%)	131 (18.3%)	303 (14.8%)	
Postgraduate	636 (9.6%)	225 (10.5%)	134 (7.7%)	56 (7.8%)	221 (10.8%)	

^a^Mean (SD); *n* (%), ^b^Kruskal-Wallis test; chi-square test of independence.

**Figure 1:**
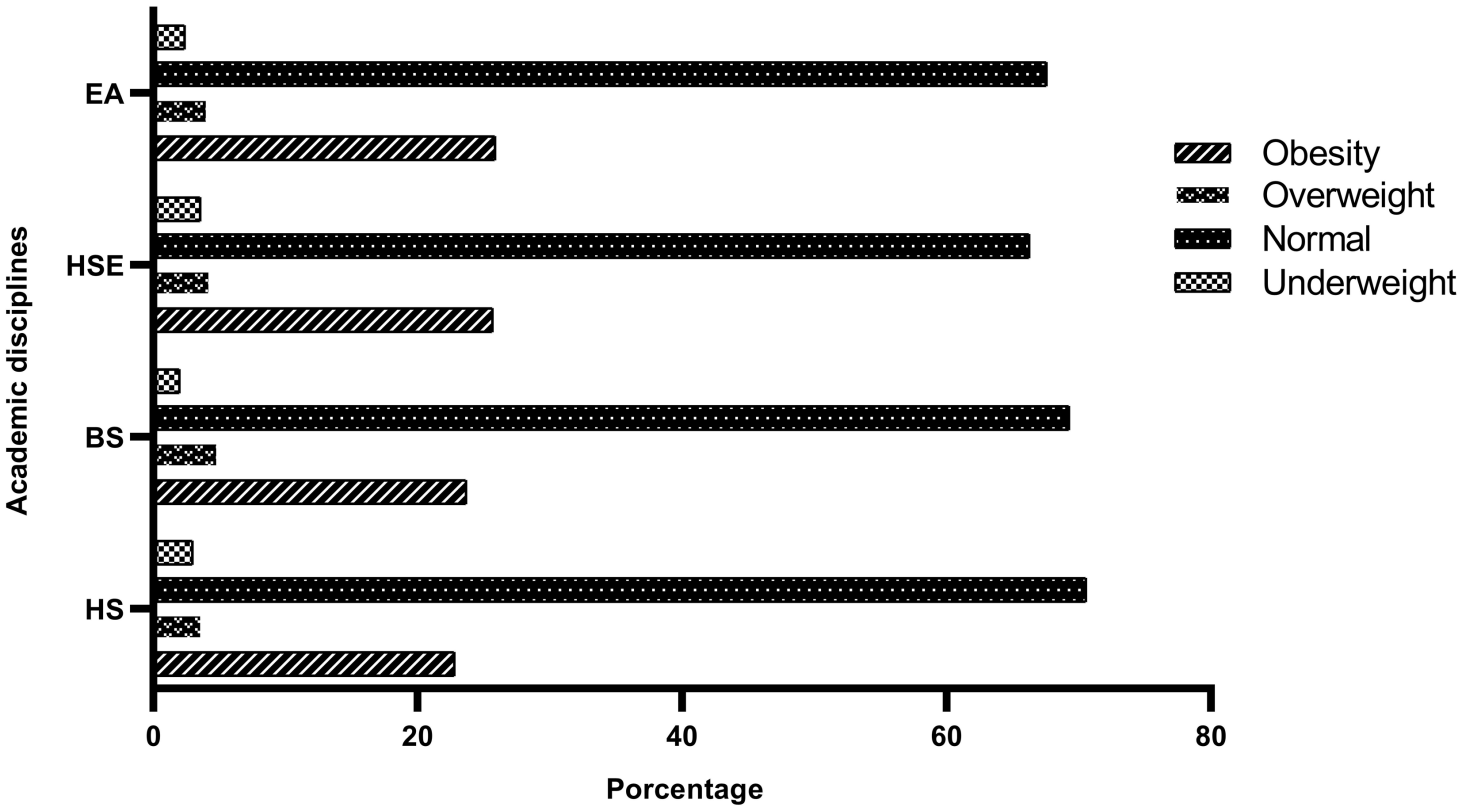
Percentage of BMI categories according to academic discipline. HS, Health Sciences; BS, Business Sciences; HSE, Human Sciences and Education; EA, Engineering/Architecture.

**Figure 2:**
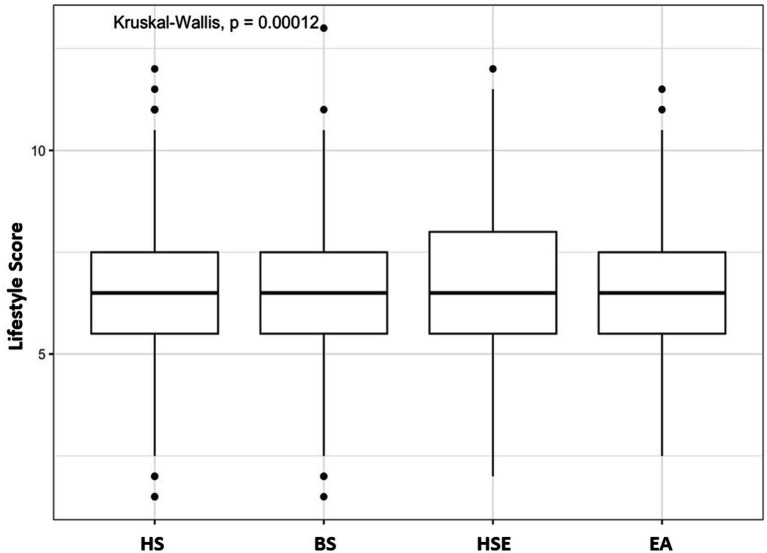
Low and whisker plot of lifestyle scores according to study disciplines. HS, Health Sciences; BS, Business Sciences; HSE, Human Sciences and Education; EA, Engineering/Architecture; Kruskal-Wallis, *p* = 0.00012.

The lowest BMI was observed in students of Health Sciences students (23.5, *p* = 0.004; Figure 1). Engineering/Architecture discipline was associated with excess body weight (overweight/obesity; *p* = 0.040). The Healthy lifestyle scale score was significantly lower in Business Science students (6.44, *p* < 0.001; Figure 2; Table 2).

**Table 2:** Anthropometric parameters and healthy lifestyle according to academic discipline in Peruvian university students.

Characteristics	All	Academic disciplines				
	*N* = 6,642^a^	Health Sciences	Business Sciences	Human Sciences and Education	Engineering/Architecture	*p* ^b^
		*N* = 2,135^a^ (32.1%)	*N* = 1,748^a^ (26.3%)	*N* = 715^a^ (10.8%)	*N* = 2,044 ^a^ (30.8%)	
Weight	63 (11)	60 (10)	63 (11)	63 (11)	65 (11)	<0.001
Height	1.62 (0.08)	1.60 (0.08)	1.62 (0.08)	1.62 (0.09)	1.65 (0.08)	<0.001
BMI	23.8 (3.2)	23.5 (3.1)	23.9 (3.2)	23.7 (3.2)	23.9 (3.3)	0.004
BMI categorized						0.040
Underweight	173 (2.6%)	63 (3.0%)	35 (2.0%)	26 (3.6%)	49 (2.4%)	
Normal	4,574 (68.9%)	1,507 (70.6%)	1,212 (69.3%)	474 (66.3%)	1,381 (67.6%)	
Obesity	279 (4.2%)	78 (3.7%)	86 (4.9%)	31 (4.3%)	84 (4.1%)	
Overweight	1,616 (24.3%)	487 (22.8%)	415 (23.7%)	184 (25.7%)	530 (25.9%)	
Healthy lifestyle	6.51 (1.50)	6.55 (1.51)	6.44 (1.50)	6.73 (1.57)	6.46 (1.47)	<0.001

^a^Mean (SD); *n* (%); % according to faculty add up to 100% per column, ^b^Kruskal-Wallis test; Chi-square test of independence, BMI, Body mass index.

In relation to the dietary component of healthy lifestyle practices, in general, the highest proportion of students had low consumption of whole grains, vegetables, fruits, nuts, and seeds. Furthermore, low levels of physical activity, water consumption, and adequate sunlight exposure were observed. Specifically, considering the recommended intake of different food groups, Business Studies and Engineering/Architecture were significantly associated with a low consumption of legumes (<1 serving/day; *p* < 0.001). Low intake of nuts and seeds (<4 servings/week; *p* < 0.001) was associated with Entrepreneurial Sciences. High dairy intake (>2 servings/day; *p* = 0.029) is associated with Business Science and Engineering/Architecture. Human Sciences and Education students reported higher consumption of sweets (>5 servings/week; *p* < 0.001) and lower intake of foods sources of vitamin B-12 (<1.0 mcg serving equivalent/day; *p* = 0.010). Business Science students consumed meat more frequently (>1 time/week; *p* < 0.001) and reported low water intake (<4 glasses of water/day, *p* < 0.001). There was no significant association with the consumption of whole grains, vegetables, fruits, eggs, regular physical activity, and adequate sunlight exposure (*p* > 0.05; Table 3).

**Table 3:** Healthy lifestyle practices according to academic discipline in Peruvian university students.

Items of healthy lifestyle practices	All	Academic disciplines				
	*N* = 6,6,42*1*	Health Sciences	Business Sciences	Human Sciences and Education	Engineering/Architecture	*p* ^b^
		*N* = 2,135^a^ (32.1%)	*N* = 1,748^a^ (26.3%)	*N* = 715^a^ (10.8%)	*N* = 2,044 ^a^ (30.8%)	
Whole grains						0.470
<3 servings/day	3,840 (57.8%)	1,264 (59.2%)	1,022 (58.5%)	405 (56.6%)	1,149 (56.2%)	
≥3 and <6 servings/day	2,409 (36.3%)	755 (35.4%)	621 (35.5%)	262 (36.6%)	771 (37.7%)	
≥6 servings/day	393 (5.9%)	116 (5.4%)	105 (6.0%)	48 (6.7%)	124 (6.1%)	
Legumes, soy, and meat substitutes						<0.001
<1 serving/day	2,550 (38.4%)	757 (35.5%)	746 (42.7%)	247 (34.5%)	800 (39.1%)	
≥1 and <3 servings/day	3,388 (51.0%)	1,149 (53.8%)	824 (47.1%)	366 (51.2%)	1,049 (51.3%)	
≥3 servings/day	704 (10.6%)	229 (10.7%)	178 (10.2%)	102 (14.3%)	195 (9.5%)	
Vegetables						0.120
<4 servings/day	3,077 (46.3%)	985 (46.1%)	851 (48.7%)	332 (46.4%)	909 (44.5%)	
≥4 and <8 servings/day	2,955 (44.5%)	948 (44.4%)	730 (41.8%)	320 (44.8%)	957 (46.8%)	
≥8 servings/day	610 (9.2%)	202 (9.5%)	167 (9.6%)	63 (8.8%)	178 (8.7%)	
Fruits						0.642
<2 servings/day	2,398 (36.1%)	763 (35.7%)	639 (36.6%)	261 (36.5%)	735 (36.0%)	
≥2 and <4 servings/day	3,134 (47.2%)	989 (46.3%)	821 (47.0%)	337 (47.1%)	987 (48.3%)	
≥4 servings/day	1,110 (16.7%)	383 (17.9%)	288 (16.5%)	117 (16.4%)	322 (15.8%)	
Nuts and seeds						<0.001
<4 servings/week	3,933 (59.2%)	1,285 (60.2%)	1,069 (61.2%)	407 (56.9%)	1,172 (57.3%)	
≥4 servings/week and <1.5 servings/day	1,917 (28.9%)	600 (28.1%)	482 (27.6%)	193 (27.0%)	642 (31.4%)	
≥1.5 servings/day	792 (11.9%)	250 (11.7%)	197 (11.3%)	115 (16.1%)	230 (11.3%)	
Vegetable oils						0.002
>4 servings/day	357 (5.4%)	88 (4.1%)	100 (5.7%)	48 (6.7%)	121 (5.9%)	
>2 and ≤4 servings/day	1,823 (27.4%)	574 (26.9%)	466 (26.7%)	175 (24.5%)	608 (29.7%)	
≤2 servings/day	4,462 (67.2%)	1,473 (69.0%)	1,182 (67.6%)	492 (68.8%)	1,315 (64.3%)	
Dairy products						0.029
>2 servings/day	745 (11.2%)	208 (9.7%)	209 (12.0%)	81 (11.3%)	247 (12.1%)	
>0 and ≤2 servings/day	4,189 (63.1%)	1,372 (64.3%)	1,098 (62.8%)	423 (59.2%)	1,296 (63.4%)	
0 ration/day	1,708 (25.7%)	555 (26.0%)	441 (25.2%)	211 (29.5%)	501 (24.5%)	
Eggs						0.059
>1 serving/day	1,157 (17.4%)	371 (17.4%)	331 (18.9%)	124 (17.3%)	331 (16.2%)	
>0 and ≤1 serving/day	4,389 (66.1%)	1,445 (67.7%)	1,133 (64.8%)	460 (64.3%)	1,351 (66.1%)	
0 serving/day	1,096 (16.5%)	319 (14.9%)	284 (16.2%)	131 (18.3%)	362 (17.7%)	
Sweets						<0.001
>5 servings/week	461 (6.9%)	137 (6.4%)	116 (6.6%)	57 (8.0%)	151 (7.4%)	
>2 and ≤5 servings/week	2,339 (35.2%)	669 (31.3%)	634 (36.3%)	213 (29.8%)	823 (40.3%)	
0–2 servings/week	3,842 (57.8%)	1,329 (62.3%)	998 (57.1%)	445 (62.2%)	1,070 (52.3%)	
Reliable sources of vitamin B-12						0.010
<1.0 mcg serving equivalent/day	2,152 (32.4%)	686 (32.1%)	546 (31.2%)	264 (36.9%)	656 (32.1%)	
≥1.0 and <2.0 mcg serving equivalent/day	1,268 (19.1%)	448 (21.0%)	329 (18.8%)	111 (15.5%)	380 (18.6%)	
≥2.0 mcg serving equivalent/day	3,222 (48.5%)	1,001 (46.9%)	873 (49.9%)	340 (47.6%)	1,008 (49.3%)	
Flesh-food intake						<0.001
>1 time/week	2,696 (40.6%)	896 (42.0%)	742 (42.4%)	244 (34.1%)	814 (39.8%)	
>1 time/month and ≤1 time/week	2,942 (44.3%)	903 (42.3%)	779 (44.6%)	325 (45.5%)	935 (45.7%)	
≤1 time/month	1,004 (15.1%)	336 (15.7%)	227 (13.0%)	146 (20.4%)	295 (14.4%)	
Daily exercise						0.632
0 min/day of any moderate or vigorous exercise	1,641 (24.7%)	522 (24.4%)	445 (25.5%)	159 (22.2%)	515 (25.2%)	
>0 and <30 min/day of moderate exercise or >0 and <15 min/day of vigorous exercise	3,588 (54.0%)	1,162 (54.4%)	924 (52.9%)	394 (55.1%)	1,108 (54.2%)	
≥30 min/day of moderate exercise or ≥15 min/day of vigorous exercise	1,413 (21.3%)	451 (21.1%)	379 (21.7%)	162 (22.7%)	421 (20.6%)	
Water intake						<0.001
<4 glasses of water/day	2,104 (31.7%)	682 (31.9%)	563 (32.2%)	203 (28.4%)	656 (32.1%)	
≥4 and <8 glasses of water/day	3,292 (49.6%)	1,007 (47.2%)	891 (51.0%)	336 (47.0%)	1,058 (51.8%)	
≥8 glasses of water/day	1,246 (18.8%)	446 (20.9%)	294 (16.8%)	176 (24.6%)	330 (16.1%)	
Sunlight exposure						0.235
<5 min/day	2,274 (34.2%)	764 (35.8%)	563 (32.2%)	243 (34.0%)	704 (34.4%)	
≥5 and <10 min/day	3,038 (45.7%)	967 (45.3%)	805 (46.1%)	325 (45.5%)	941 (46.0%)	
≥10 min/day	1,330 (20.0%)	404 (18.9%)	380 (21.7%)	147 (20.6%)	399 (19.5%)	

^a^*n* (%), ^b^chi-square test of independence.

In Table 4, students in Business Sciences (B = −0.11, 95% CI: −0.20 to −0.01; *p* = 0.024) and Engineering/Architecture (B = −0.09, 95% CI: −0.18 to −0.00; *p* = 0.047) had a lower mean score on the measure of healthy lifestyle compared to those in Health Sciences; in contrast, students in Human Sciences and Education had the highest average score. Similarly, students enrolled in Business Sciences (B = 0.35, 95% CI: 0.15–0.56; *p* = 0.001) and Engineering/Architecture (B = 0.32, 95% CI: 0.13–0.52; *p* = 0.001) had a significantly higher average BMI than those in Health Sciences. The coefficients remained in the same direction after adjusting for age, sex, and parental education.

**Table 4:** Simple and multiple regression models between academic discipline and lifestyle and anthropometric parameters.

	Simple regression			Multiple regression^a^		
Academic disciplines	B	95% CI	*p*	B	95% CI	*p*
	**Healthy lifestyle practices**					
Health sciences	Ref.			Ref.		
Business Sciences	−0.11	−0.20 – −0.01	0.024	−0.15	−0.24 – −0.05	0.003
Human Sciences and Education	0.18	0.06–0.31	0.005	0.14	0.01–0.27	0.037
Engineering/Architecture	−0.09	−0.18 – −0.00	0.047	−0.22	−0.31 – −0.12	<0.001
	**BMI**					
Health sciences	Ref.			Ref.		
Business Sciences	0.35	0.15–0.56	0.001	0.33	0.12–0.53	0.002
Human Sciences and Education	0.19	−0.08 – 0.46	0.161	0.02	−0.25 – 0.29	0.874
Engineering/Architecture	0.32	0.13–0.52	0.001	0.36	0.15–0.56	0.001

^a^Adjusted for age group, sex, and parental education. BMI, Body mass index.

## Discussion

The transition to university life represents a critical period in the development of habits and behaviors that can have a lasting impact on students’ health status. In this context, lifestyle plays an important role, especially regarding the consumption of plant-based foods, regular physical activity, among others. Eating behaviors, along with other aspects of lifestyle, are influenced by a variety of factors, including the student’s field of study. The present study compared BMI and healthy lifestyle practices, considering specific dietary behaviors, such as the intake of whole grains, legumes, vegetables, fruits, nuts, and seeds among university students from different academic areas including Health Sciences, Business Sciences, Human Sciences and Education, and Engineering/Architecture. This research seeks to understand how academic disciplines, with their specific demands, can influence the life habits of students.

### BMI

Although obesity commonly manifests itself in the early stages, university students also go through a worrisome phase in which their lifestyle can be subjected to unhealthy changes, often resulting in weight gain (14). The probability of being overweight or obese is approximately the same among young men and women (40). However, research on university students shows an increasing trend of obesity among men (4, 5).

In the current study, regression analysis found that Business and Engineering/Architecture students were more likely to report excess body weight than Health Sciences students, who had the lowest mean BMI score. Similarly, a comparable study conducted with 584 participants during the COVID-19 pandemic reported that students in Science and Technology disciplines had a higher proportion of individuals with overweight and obesity compared to those in Health Sciences (10). Furthermore, in our study, a higher prevalence of individuals with excess body weight (28.5%) and a lower prevalence of underweight individuals (2.6%) were found. These findings are consistent with studies conducted in the university population (34). Differences in the prevalence of excess weight among students from different disciplines can be attributed to factors such as academic stress, irregular schedules, academic workload, sedentary lifestyle, and health and nutrition education (6). In fact, academic stressors can cause systemic imbalance, affecting both the physical and psychological well-being of students, and leading to behaviors that can increase the risk of obesity (41). Therefore, the current study highlights the need to address the lifestyles and eating habits of university students, considering the particularities of each discipline of study. Promoting strategies to manage BMI-related issues could be a key approach to prevent the development of obesity-related chronic diseases in the long term.

### Healthy lifestyle practices

#### Adequate food intake

Consuming of whole grains, legumes, vegetables, fruits, nuts, and seeds offers multiple health benefits. For example, whole grains improve digestion and reduce the risk of chronic diseases (42). In the case of legumes, they provide protein and fiber and are beneficial for weight control and cardiovascular health (43). Vegetables and fruits, rich in vitamins, minerals, and antioxidants, reduce the risk of heart disease and cancer (44). Nuts and seeds, sources of healthy fats and proteins, contribute to cardiovascular health and cholesterol control (45). Although these foods are essential for a balanced diet, positively impacting health and prevention of noncommunicable diseases, in the current study, in general, the highest proportion of students had a low consumption of whole grains, vegetables, fruits, nuts, and seeds. Specifically, according to the results of the regression analysis, students in Business and Engineering/Architecture had a lower average lifestyle score compared to those in the Health Sciences faculty. In addition, the students in Human Sciences and Education had the highest healthy lifestyle score.

These findings are similar to those found in previous studies conducted in university students in several countries. In a recent study of Saudi Arabian university students, only 16.07% and 11.23% of 454 students consumed vegetables and fruits daily, respectively (14). Furthermore, it was found that only a small proportion of Thai university students consumed vegetables and fruits at the recommended levels (10). Similarly, among Turkish university students, it was found that 66.1% of men and 63.1% of women had insufficient consumption of fruits and vegetables (46). Additionally, it was found that the dietary habits of Spanish students were poor in terms of legume intake, showing that 75.8% had inadequate consumption (≤2 times/week) of legumes (47). Regarding the consumption of nuts and seeds, our study is consistent with the findings of research that demonstrated legumes, nuts, and seeds were the least consumed food groups among students (48). Given the importance of consuming these foods for health, it is suggested that strategies be implemented to increase their intake among university students.

Likewise, the current study revealed significant differences in consumption patterns among students in various academic disciplines. Compared to Health Sciences students, it was found that students from Business Sciences, Engineering/Architecture, and Humanities and Education faculties are associated with a higher consumption of dairy products, exceedingly more than 2 portions per day. Moreover, egg consumption exceeded 1 portion daily, while the intake of sweets was greater than 5 portions per week. Students may be unaware of the health impacts associated with excessive consumption of added sugar (49). Likewise, other studies carried out in university students have reported similar findings. For example, the results of a cross-sectional study in university students indicated that 45.8% consumed sweets daily (50). Also, the prevalence of daily consumption of sugar-sweetened beverages and sugar-sweetened fruit in the last month and daily was 91 and 50%, respectively (51). Likewise, in the current study, meat consumption was frequent, exceeding 1 time per week. These findings are similar to a study that reported that 47.1% of Science and Technology students consumed meats almost every day (10). These consumption patterns contrast with the general recommendations for a balanced and healthy diet, which suggest a moderate intake of dairy products and eggs, limiting the consumption of sweets and meat, especially processed meats (52). These findings highlight the need to promote nutritional education among university students, regardless of their field of study, to encourage healthy eating habits.

#### Adequate water intake

Water is the main chemical component of the body, accounting for approximately 50 to 70% of body weight (53). Scientific evidence and current daily water intake recommendations from national organizations, such as the Ministry of Health of Peru, Institutes of Medicine, and the European Food Safety Authority, agree that for optimal health, it is important to consume between 8 to 12 glasses of water a day (53–55). However, low water intake is one of the most common health concerns affecting both the general population and university students, with the latter being especially more susceptible (25–27). In the current study, only 18.58% of students reported meeting these water intake recommendations. Specifically, Engineering/Architecture and Business Sciences students reported the lowest proportions of adequate water intake, at 16.1% and 16.8%, respectively.

Previous studies have shown similar trends in low water intake among university students (25–27, 56). For example, a study of U.S. university students during the COVID-19 pandemic found that only 16.3% of women and 13.3% of men consumed the recommended amount of water daily (25). Another study conducted in Iranian university students reported that the average daily fluid intake of individuals, especially water, was below the recommended values (27). Similarly, the results of the current study are consistent with the findings of a previous study in Turkish university students, where it was reported that only 19% met their water needs with drinking water recommended for adults (26). These findings are concerning, considering the importance of water for numerous physiological functions in the body, including regulating body temperature, transporting nutrients, and eliminating waste (28). Insufficient water intake can lead to dehydration, affecting cognition, physical performance, and general well-being of university students (56). While the exact reasons for these findings cannot be determined with complete accuracy, they could be attributed to a lack of awareness about hydration needs, a preference for other beverages, busy lifestyles, and limited access to drinking water throughout the day. All these factors could contribute to insufficient water intake in this population. However, we cannot provide a comprehensive explanation for the reduction in water intake previously discussed. Nonetheless, the current results are in line with several previous studies, underscoring the need to promote greater awareness about the importance of adequate hydration among university students. Universities could play an important role in this regard by implementing hydration education programs and ensuring easy access to drinking water sources on campuses. Additionally, it would be beneficial to integrate public health messages on hydration into student wellness campaigns, given the clear need to improve water intake habits among students, especially in fields like Engineering/Architecture and Business Sciences.

#### Regular physical activity

Sedentary lifestyles can negatively impact students’ health, increasing the risk of non-communicable diseases, such as obesity, heart disease and diabetes, and poor academic performance (10, 23). Numerous studies have confirmed the importance of physical exercise in promoting health, constituting an essential component in global intervention strategies, integrating health policies in developed and developing countries (10, 21, 57, 58). In the current study, the results indicate less physical activity and align with trends observed in previous studies (10, 19–21). For example, Arias-Palencia et al. (21) demonstrated that most Spanish university students engaged in less physical exercise than recommended. Additionally, several studies conducted during COVID-19 confinement revealed a reduction in physical exercise among young people, especially among university students (10, 19, 20). It is important to note that the WHO recommend a minimum of 150 min of moderate physical activity per week for individuals between 18 and 67 years of age (23). Furthermore, in our study, although there were no significant differences, students from Human Sciences and Education and Health Sciences, reported slightly higher levels of moderate and vigorous physical exercise, compared to Business Sciences and Engineering/Architecture. This aligns with a study conducted with German university students that measured physical activity in terms of metabolic equivalent of task (MET) minutes per week. It showed that students in Natural Sciences, Mathematics, and Computer Science, with 3,427 MET-minutes per week, and those in Language, Humanities, and Cultural Studies, with 3,553 MET-minutes per week, reported the lowest levels of physical activity. On the other hand, students in Education (4,312 MET-minutes per week), Medicine (3,981 MET-minutes per week), and Social Sciences, Communication, and Sports (3,844 MET-minutes per week) recorded the highest levels of physical activity (58). Contrary findings were observed in a study that demonstrated that, although Health Sciences students possessed medical knowledge, their adherence to physical exercise recommendations turned out to be like students from other disciplines (10). This indicates that health knowledge does not necessarily translate into increased physical exercise among these students. The reduction and differences in physical activity among students from different disciplines may be due to multiple causes, including an increase in academic and social obligations, changes in their environment and lifestyle, as well as limited time or resources. This understanding is important for developing personalized strategies to promote physical exercise among the general student population, regardless of their field of study.

#### Adequate sunlight exposure

Sunlight is essential for the physical health and mental well-being of individuals (29). Both observational and experimental evidence has consistently reaffirmed the positive effects of sunlight exposure (59); these include the prevention and treatment of various dermatological conditions, such as psoriasis and eczema (29). Sunlight acts therapeutically on these skin disorders, improving symptoms and the quality of life of those affected (60). In addition, sunlight exposure is essential for the photosynthesis of vitamin D in the skin, a process essential for the maintenance of bone and muscle health (61). Vitamin D, synthesized through sun exposure, plays a significant role in regulating calcium and phosphorus, key elements for bone strength and development (60). In our study, we found that only 20.0% of students reported compliance with sunlight exposure recommendations. Specifically, students from Health Sciences and Engineering/Architecture reported the lowest proportions of sunlight exposure, with 18.9 and 19.5%, respectively. This finding is consistent with previous studies that have also reported low sunlight exposure in university populations. For example, a study found that many university students in the United Arab Emirates did not receive sufficient sunlight, which could pose a public health problem due to a potential vitamin D deficiency (32). Additionally, a similar study found low sun exposure practices among Sri Lankan university students in healthcare studies (30), aligning with our findings. Moreover, other studies have highlighted how modern urban lifestyles, common among university students, limit sunlight exposure (31). This is exacerbated by technology and preference for indoor activities, which further reduces opportunities for exposure to natural sunlight (62, 63). On the other hand, a lack of knowledge about vitamin D, long hours spent in academic facilities, and sedentary study habits are some of the reasons for low sunlight exposure (30, 32); this is particularly relevant for Health Sciences and Engineering/Architecture students, whose rigorous academic programs often involve a greater amount of time spent indoors. The low sunlight exposure in these groups of university students suggests the need to encourage outdoor activities. Universities could design academic schedules that allow for outdoor breaks or promote extracurricular activities that occur in outdoor environments. However, it is important to balance sun exposure with the risk of skin damage, including skin cancer (33), which implies the need for public health strategies that promote a balance between obtaining benefits and minimizing the risks associated with sun exposure.

### Limitations and future research

One of the key limitations of this study lies in its conduct at a university affiliated with the Seventh-day Adventist Church, a denomination known for promoting healthy lifestyle practices among its members, including specific diets and eating patterns. This orientation toward healthy lifestyle habits could influence student behavior patterns, regardless of their personal religious belief as the institution philosophy can indirectly promote certain dietary and health practices among the entire student community. Although there is a diverse representation of religious beliefs at the university, the influence of Adventist philosophy in the university environment may have contributed to some homogenization of students’ lifestyle practices, thus limiting the generalizability of our findings to populations with different religious and cultural backgrounds. Future studies could include a more diverse sample of adults and children from other geographic areas to examine BMI and lifestyle practices. On the other hand, it is important to note that the study did not consider other relevant aspects of lifestyle, such as alcohol and tobacco consumption and adequate rest; the omission of these factors widely recognized for their impact on health can limit understanding of how various lifestyle elements interact with study disciplines in university students. Therefore, future research should include these factors to provide a more complete and nuanced analysis of the lifestyle and field of study. Another significant limitation of this study is its cross-sectional nature, which implies that it cannot provide information on how the phenomena studied develop over time or establish causal relationships between variables, which may also limit the generalizability of the findings, as it only provides a snapshot relationship at a specific point in time without considering the evolution or change of behaviors and attitudes over time. Longitudinal studies are suggested to better understand how BMI and lifestyle of students evolve over time. Moreover, it is important to mention that the weight and height of the participants were self-reported. People tend to underestimate their weight and overestimate their height, which could introduce significant errors and biases in the data collected. Anthropometric data were collected in this manner due to restrictions imposed in the context of the COVID-19 pandemic. Additionally, lifestyle practices were based on self-reported reports, which could cause response biases, as participants may have difficulty accurately recalling their lifestyle habits or may tend to present a more favorable image of their behavior. Consequently, it is important that future research employ more objective and accurate methods to collect data on lifestyle patterns. Finally, we acknowledge participant self-selection as an inherent limitation of our study. It is possible that those individuals who chose to respond to our survey were motivated, in part, by their healthy lifestyle. This may introduce a bias in our sample, as students with more health-conscious practices may be overrepresented compared to those whose lifestyle habits are less healthy. Such a self-selection bias limits the ability to generalize our findings to the entire university student population. In future research, it would be beneficial to implement strategies that encourage the participation of a more representative sample of the diversity of lifestyles present in the university community.

### Public health implications

Despite the limitations of the current study, we believe that the results obtained are of significant relevance, especially in the context of the formulation of public health and educational policy. These findings provide comprehensive formulation on the lifestyles of a young, academically educated population, which is important, as it often sets guidelines for behaviors and habits that last throughout life. This detailed understanding can be invaluable in developing targeted strategies to promote healthy habits in the early stages, which have the potential to positively influence long-term health and well-being. These strategies could include the promotion of nutrition education, regardless of their field of study, to encourage healthy eating habits; integration of public health messages on hydration in student wellness campaigns, due to the need to improve water intake habits among students; development of individualized interventions to promote physical activity among the student population; and the design of academic schedules that favor active outdoor breaks and the promotion of extracurricular activities that occur in outdoor environments.

## Conclusion

In summary, this study reveals that students belonging to areas such as Business Sciences and Engineering/Architecture have a higher BMI compared to those in the Health Sciences field. In addition, these groups tend to lean toward less healthy lifestyles. In general, it was observed that the students reported insufficient consumption of foods such as whole grains, legumes, vegetables, nuts, and seeds. Likewise, the levels of regular physical activity, adequate hydration, and adequate sunlight exposure were low. Although the students of Human Sciences and Education and Health Sciences exhibited healthier eating patterns and lifestyles, there is a clear need to improve eating and living habits in general among the university student population to mitigate the risk factors associated with non-communicable diseases.

## Data availability statement

The raw data supporting the conclusions of this article will be made available by the authors, without undue reservation.

## Ethics statement

The studies involving humans were approved by Research Ethics Committee of the Universidad Peruana Unión (approval number: 2021-CEUPeU-0009). The studies were conducted in accordance with the local legislation and institutional requirements. The participants provided their written informed consent to participate in this study.

## Author contributions

JS: Conceptualization, Validation, Visualization, Writing – original draft, Writing – review & editing. YC-M: Conceptualization, Methodology, Visualization, Writing – original draft, Writing – review & editing. SC-C: Investigation, Project administration, Writing – review & editing. AS-B: Data curation, Investigation, Writing – review & editing. CR-V: Data curation, Investigation, Resources, Writing – review & editing. SO-G: Data curation, Investigation, Resources, Writing – review & editing.
