# Supplementary material for: Body mass index and healthy lifestyle practices among Peruvian university students: a comparative study among academic discipline
Source: Front Nutr. 2024 Feb 21;11:1361394. doi: 10.3389/fnut.2024.1361394 (PMC10915028; doi:10.3389/fnut.2024.1361394)
Supplement: Supplementary file 1 [file Data_Sheet_1.PDF]

Appendix A. Diet and Healthy Lifestyle Scale (DEVS)–Spanish version.

1. ¿Cuántas porciones de **granos integrales** consume en un día? ***(pan integral, avena, quínoa, arroz o trigo integral, etc.). Una porción equivale a*** *los siguientes ejemplos: 1 rebanada de pan de molde integral****,*** *o 4 galletitas integrales****,*** *o ½ taza mediana de trigo, quínoa o arroz integral cocidos****,*** *o 1 plato chico de pastas integrales cocidas****,*** *o ½ taza mediana de avena.*

___Menos de 3 porciones por día
___De 3 a menos de 6 porciones por día
___6 o más porciones por día

2. ¿Cuántas porciones de **legumbres y sus derivados** consumen en un día? *(lentejas, arvejas, habas, tarwi o chocho, garbanzo, soja y derivados como leche de soja, tofu, milanesas o hamburguesas de legumbres, etc.).* ***Una porción equivale a*** *los siguientes ejemplos: ½ taza mediana de lentejas, porotos, soja, garbanzos o arvejas cocidos, o 1 milanesa de legumbres grande, o 3 rebanadas de tofu, o 4 cucharadas soperas de aderezo de legumbres (ej. humus), o 1 vaso de leche de soja o 2 cucharadas soperas de leche de soja en polvo.*

___Menos de 1 porción por día
___De 1 a menos de 3 porciones por día
___3 o más porciones por día

3. ¿Cuántas porciones de **verduras** come en un día? ***(verduras crudas, verduras cocidas y jugos de verduras 100% naturales)***. ***Una porción equivale a*** *los siguientes ejemplos: 1 plato grande de verduras de hojas verdes crudas como lechuga, espinaca, etc., ó 1 cucharón pequeño de verduras crudas como tomate, zanahoria, rabanitos, cebolla, etc., ó 1 cucharón de verduras cocidas utilizadas en sopas, guisos, ensalada rusa, en tartas o empanadas, etc., ó 1 vaso mediano de jugos de verduras 100% naturales (espinaca, zanahoria, pepino, etc.)*

___Menos de 4 porciones por día
___De 4 a menos de 8 porciones por día
___8 o más porciones por día

4. ¿Cuántas porciones de frutas come en un día? ***(frutas frescas, deshidratadas, enlatadas, cocidas y jugos de frutas 100% naturales)***. ***Una porción equivale a*** *los siguientes ejemplos: 1 fruta mediana o 2 frutas pequeñas, o 2 cucharadas soperas de pasas o 1 orejón de pera o durazno, o 1 vaso mediano de jugos 100% naturales.*

___Menos de 2 porciones por día
___De 2 a menos de 4 porciones por día
___4 o más porciones por día

5. ¿Cuántas porciones de **frutos secos y semillas** consume en un día? ***(nueces, almendras, castañas, lino, chía, girasol, sésamo, etc.). Una porción equivale a*** *los siguientes ejemplos: 2 cucharadas soperas de semillas, o 10 unidades de almendras, nueces o castañas, o 1 vaso de leche o jugo de frutos secos o semillas.*

___Menos de 4 porciones por semana
___De 4 porciones por semana a 1 porción por día
___1 ½ o más porciones por día

6. ¿Cuántas porciones de **aceites vegetales no calentados** (aceite de oliva, girasol, maíz o soja, etc.), **palta y aceitunas** consume en un día? **Una porción equivale a** los siguientes ejemplos: 2 cucharadas chicas (tipo postre) de aceite no calentado o usado en la cocción, o ½ palta chica, o 3 cucharadas tipo postre de pasta de aceitunas, o 20 aceitunas enteras.

___Hasta 2 porciones por día
___Más de 2 hasta 4 porciones por día
___Más de 4 porciones por día

7. ¿Cuántas porciones de **lácteos** consume en un día? *(Queso, yogur, leche, postres lácteos, etc.)* ***Una porción equivale a*** *los siguientes ejemplos: 1 taza grande de leche o yogur, o 1 rebanada mediana de queso fresco, o 3 cucharadas soperas de queso untable.*

___No consumo
___Hasta 2 porciones por día

___Más de 2 porciones por día

8. ¿Cuántas porciones de **huevo** consume en un día? *(hervido, en preparaciones como revuelto, rellenos, tortilla, ensalada, tortas etc.)* ***Una porción equivale a*** *los siguientes ejemplos: 1 huevo o 2 claras.*

___No consumo
___Hasta 1 porción por día
___Más de 1 porción por día

9. ¿Cuántas porciones de **dulces** consume en **una semana**? *(tortas, helados, chocolates, mermeladas, dulces, bebidas azucaradas, etc.)* ***Una porción equivale a*** *los siguientes ejemplos: 1 porción de torta, o 1 cucharada tipo postre de mermelada, o 2 bochas de helado, o 6 cuadraditos de chocolate, o 1 alfajor, o 1 vaso mediano de gaseosa u otra bebida azucarada.*

___Menos de 2 porciones por semana
___De 2 a 5 porciones por semana
___Más de 5 porciones por semana

10. ¿Cuántas porciones de **fuentes confiables de vitamina B12** consume en un día? *Incluye: carne, pescado, lácteos, huevos, alimentos fortificados y suplementos.* ***Una porción equivale a*** *los siguientes ejemplos: 1 porción chica de carne (vacuna, ave y/o pescado), o ½ vaso de leche, o 2 rebanadas de queso, o 1 huevo, o 1 vaso de jugo o leche vegetal comercial fortificada con vitamina B-12, o 1 suplemento de 100 microgramos de vitamina B-12 por día, o 1 suplemento de 2000 microgramos de vitamina B-12 por semana que equivale al consumo de 2 o más porciones de suplemento de B12 por día.*

___Menos de 1 porción por día
___1 porción por día
___2 o más porciones por día

11. ¿Cuántas veces en la semana consume **carnes**? *(carne roja, pescado, pollo y carnes procesadas como chorizo, hamburguesa, salchicha, etc.)*

___No consumo
___Menos de 1 vez por mes hasta 1 vez por semana
___Más de 1 vez por semana

12. ¿Cuántos **minutos** realiza **de actividad física** en un día? *(s**i no realiza todos los días** actividad física,* ***promedie*** *en un día la actividad semanal que realice).* ***Ejemplos de actividad física (AF) moderada****: Caminata rápida, jardinería o tareas domésticas activas y trabajos de construcción generales.* ***Ejemplos de actividad física (AF) intensa****: Correr o trotar, actividades en el gimnasio, desplazamientos rápidos en bicicleta y deportes competitivos.*

___No realizo actividad física
___Menos de 30 min por día de AF moderada o menos de 15 min por día de AF intensa
___30 min o más por día de AF moderada o 15 min o más por día de AF intensa

13. ¿Cuántos **vasos de agua** de 250ml consume al día?
___Menos de 4 vasos por día
___De 4 a 7 vasos por día
___8 o más vasos por día

14. ¿Cuántos **minutos** se expone al **sol** (al menos brazos y/o piernas) diariamente **entre las 11 y las 13 hs.**?
___Menos de 5 min por día
___De 5 a menos de 10 min por día
___10 min o más por día

Cuestionario de alimentos ricos en grasas

1. ¿Aproximadamente con qué frecuencia come Hamburguesas, carne molida, tacos?

___1 vez al mes o menos = 0 punto
___de 2 a 3 veces al mes = 1 punto
___1 a 2 veces a la semana = 2 puntos
___3 a 4 veces a la semana = 3 puntos

___de 5 a más veces a la semana = 4 puntos

2. ¿Aproximadamente con qué frecuencia come carne de res o cerdo, como filetes, asados, costillas o en Bocadillos?

___1 vez al mes o menos = 0 punto
___de 2 a 3 veces al mes = 1 punto
___1 a 2 veces a la semana = 2 puntos
___3 a 4 veces a la semana = 3 puntos
___de 5 a más veces a la semana = 4 puntos

3. ¿Aproximadamente con qué frecuencia come Pollo frito?

___1 vez al mes o menos = 0 punto
___de 2 a 3 veces al mes = 1 punto
___1 a 2 veces a la semana = 2 puntos
___3 a 4 veces a la semana = 3 puntos
___de 5 a más veces a la semana = 4 puntos

4. ¿Aproximadamente con qué frecuencia come Hot dogs o salchicha polaca o italiana?

___1 vez al mes o menos = 0 punto
___de 2 a 3 veces al mes = 1 punto
___1 a 2 veces a la semana = 2 puntos
___3 a 4 veces a la semana = 3 puntos
___de 5 a más veces a la semana = 4 puntos

5. ¿Aproximadamente con qué frecuencia come Embutidos, fiambres, jamón (no bajo en grasas)?

___1 vez al mes o menos = 0 punto
___de 2 a 3 veces al mes = 1 punto
___1 a 2 veces a la semana = 2 puntos
___3 a 4 veces a la semana = 3 puntos
___de 5 a más veces a la semana = 4 puntos

6. ¿Aproximadamente con qué frecuencia come Tocino o salchicha de desayuno?

___1 vez al mes o menos = 0 punto
___de 2 a 3 veces al mes = 1 punto
___1 a 2 veces a la semana = 2 puntos
___3 a 4 veces a la semana = 3 puntos
___de 5 a más veces a la semana = 4 puntos

7. ¿Aproximadamente con qué frecuencia come Aderezos para ensaladas (no bajos en grasa)?

___1 vez al mes o menos = 0 punto
___de 2 a 3 veces al mes = 1 punto
___1 a 2 veces a la semana = 2 puntos
___3 a 4 veces a la semana = 3 puntos
___de 5 a más veces a la semana = 4 puntos

8. ¿Aproximadamente con qué frecuencia come Margarina, mantequilla o mayonesa sobre pan o Papas?

___1 vez al mes o menos = 0 punto
___de 2 a 3 veces al mes = 1 punto
___1 a 2 veces a la semana = 2 puntos
___3 a 4 veces a la semana = 3 puntos
___de 5 a más veces a la semana = 4 puntos

9. ¿Aproximadamente con qué frecuencia come Margarina, mantequilla o aceite para cocinar Huevos (no batidos de huevos o solo claras de huevo)?

___1 vez al mes o menos = 0 punto
___de 2 a 3 veces al mes = 1 punto
___1 a 2 veces a la semana = 2 puntos

___3 a 4 veces a la semana = 3 puntos
___de 5 a más veces a la semana = 4 puntos

10. ¿Aproximadamente con qué frecuencia come Pizza?

___1 vez al mes o menos = 0 punto
___de 2 a 3 veces al mes = 1 punto
___1 a 2 veces a la semana = 2 puntos
___3 a 4 veces a la semana = 3 puntos
___de 5 a más veces a la semana = 4 puntos

11. ¿Aproximadamente con qué frecuencia come Queso, queso para untar (no bajo en grasa)?

___1 vez al mes o menos = 0 punto
___de 2 a 3 veces al mes = 1 punto
___1 a 2 veces a la semana = 2 puntos
___3 a 4 veces a la semana = 3 puntos
___de 5 a más veces a la semana = 4 puntos

12. ¿Aproximadamente con qué frecuencia come Leche entera?

___1 vez al mes o menos = 0 punto
___de 2 a 3 veces al mes = 1 punto
___1 a 2 veces a la semana = 2 puntos
___3 a 4 veces a la semana = 3 puntos
___de 5 a más veces a la semana = 4 puntos

13. ¿Aproximadamente con qué frecuencia come Papas fritas?

___1 vez al mes o menos = 0 punto
___de 2 a 3 veces al mes = 1 punto
___1 a 2 veces a la semana = 2 puntos
___3 a 4 veces a la semana = 3 puntos
___de 5 a más veces a la semana = 4 puntos

14. ¿Aproximadamente con qué frecuencia come Chips de maíz, papas fritas, palomitas de maíz, galletas saladas?

___1 vez al mes o menos = 0 punto
___de 2 a 3 veces al mes = 1 punto
___1 a 2 veces a la semana = 2 puntos
___3 a 4 veces a la semana = 3 puntos
___de 5 a más veces a la semana = 4 puntos

16. ¿Aproximadamente con qué frecuencia come Donas, pasteles, galletas (no bajas en grasa)?

___1 vez al mes o menos = 0 punto
___de 2 a 3 veces al mes = 1 punto
___1 a 2 veces a la semana = 2 puntos
___3 a 4 veces a la semana = 3 puntos
___de 5 a más veces a la semana = 4 puntos

16. ¿Aproximadamente con qué frecuencia come Helado (sin sorbete ni sin grasa)?

___1 vez al mes o menos = 0 punto
___de 2 a 3 veces al mes = 1 punto
___1 a 2 veces a la semana = 2 puntos
___3 a 4 veces a la semana = 3 puntos
___de 5 a más veces a la semana = 4 puntos
